# Retraction Note: Restoring BMP4 expression in vascular endothelial progenitors ameliorates maternal diabetes-induced apoptosis and neural tube defects

**DOI:** 10.1038/s41419-026-08781-3

**Published:** 2026-04-20

**Authors:** Songying Cao, E. Albert Reece, Wei-Bin Shen, Peixin Yang

**Affiliations:** 1 Department of Obstetrics, Gynecology & Reproductive Science, University of Maryland School of Medicine, Baltimore, MD 21201 USA; 2 Department of Biochemistry and Molecular Biology, University of Maryland School of Medicine, Baltimore, MD 21201 USA

Retraction Note to: *Cell Death & Disease* 10.1038/s41419-020-03078-5, published online 15 October 2020

The Editors-in-Chief have retracted this article. Following publication, the University of Maryland, Baltimore conducted an internal investigation into concerns raised regarding this article, specifically:

- Two panels appear to overlap in Figure 5B for the conditions representing different Non-diabetic mice, non-BMP4-Tg and BMP4-Tg.
- Images of stained embryonic yolk sacs of Flk-1+ mice overexpressing BMP4 in Figure 5D appear to overlap with images of stained embryonic yolk sacs of Flk-1+ mice overexpressing survivin in Figure 5d of another publication by the same authors [1].

The Investigation Committee recommended retraction of the article to correct the scientific record and ensure its integrity. Raw data for these images could not be authenticated by the Investigation Committee. The Editors-in-Chief have therefore lost confidence in the data and conclusions of this article.

The authors did not respond to correspondence from the publisher regarding this retraction.
